# Forecasting of the first hour aftershocks by means of the perceived magnitude

**DOI:** 10.1038/s41467-019-10763-3

**Published:** 2019-07-04

**Authors:** E. Lippiello, G. Petrillo, C. Godano, A. Tramelli, E. Papadimitriou, V. Karakostas

**Affiliations:** 1 Department of Mathematics and Physics, University of Campania “L. Vanvitelli”, Viale Lincoln, 5, 81100 Caserta, Italy; 2 Istituto Nazionale Geofisica e Vulcanologia, Sez. Napoli, Osservatorio Vesuviano, Via Diocleziano 328, Napoli, 80124 Italy; 3 Geophysics Department, Aristotle University of Thessaloniki, 54124 Thessaloniki, Greece

**Keywords:** Geophysics, Seismology

## Abstract

The majority of strong earthquakes takes place a few hours after a mainshock, promoting the interest for a real time post-seismic forecasting, which is, however, very inefficient because of the incompleteness of available catalogs. Here we present a novel method that uses, as only information, the ground velocity recorded during the first 30 min after the mainshock and does not require that signals are transferred and elaborated by operational units. The method considers the logarithm of the mainshock ground velocity, its peak value defined as the perceived magnitude and the subsequent temporal decay. We conduct a forecast test on the nine *M * ≥ 6 mainshocks that have occurred since 2013 in the Aegean area. We are able to forecast the number of aftershocks recorded during the first 3 days after each mainshock with an accuracy smaller than 18% in all cases but one with an accuracy of 36%.

## Abstract (web summary)

The timing and locations of aftershocks following the initial impact of an earthquake are key to mitigate potential further hazards. Here the authors use the seismic ground velocity as input parameter to provide accurate probabilities of post seismic occurrence within 30 min of the main shock.

## Introduction

Short-term aftershock forecasting (STAF) models^1–3^ are based on the Omori–Utsu (OU) law^4^ which states that the rate of aftershocks with magnitude *M* above a threshold *M*_*c*_, at time *t* after the mainshock, is given by

1 $$\lambda (t) = \frac{{K10^{b{\mathrm{\Delta }}M}}}{{(t + c)^p}},$$

where Δ*M* = *M*_*m*_ − *M*_*c*_ and *M*_*m*_ is the mainshock magnitude. Equation (1) may be used in STAF models once the four model parameters *b*, *p*, *K*, and *c* are known. The parameters *b* and *p* are similar for different sequences and setting *b* ≃ *p* ≃ 1 almost always provides reasonable results. Conversely the parameters *K* and *c* exhibit huge fluctuations from one sequence to another^5^ and, therefore, they must be fitted as soon as the ongoing earthquake sequence produces a sufficient number of aftershocks^3^. This fitting procedure requires the identification of all aftershocks above a sufficiently small magnitude threshold. This, however, is a very difficult task because of the overlapping of coda-waves, among close in time aftershocks, which obscures the recordings of smaller events. As a consequence small aftershocks are not recorded in the first part of seismic sequences and the evaluation of *K* and *c* is strongly biased^6–18^. Some fitting procedures^11,13,16–20^ have been recently developed to take into account this incompleteness of data sets, but they become efficient only when a sufficient number of aftershocks has been recorded. Another problem of STAF methods is that, once *λ*(*t*) has been estimated, the predicted aftershock rate must be converted in terms of the probability of ground shaking at a given site. This requires accurate empirical attenuation functions^21,22^ which are usually only approximately known and often ignore site effects caused by local site conditions. Here we present a novel method based on a fitting procedure applied to the ground velocity recorded at a site of interest. The method does not require the identification of aftershocks and provides the probability of strong ground shaking without attenuation relations. This idea is, to a certain extent, similar to the proposal^23^ of extrapolating the long-term probability of ground shaking directly from the frequency distribution of the maximum amplitude of seismograms^24^. Also in our method, indeed, it is not necessary to locate earthquakes and to rely on assumptions on seismicity.

## Results

### Data set and the envelope function

**Fig. 1:**
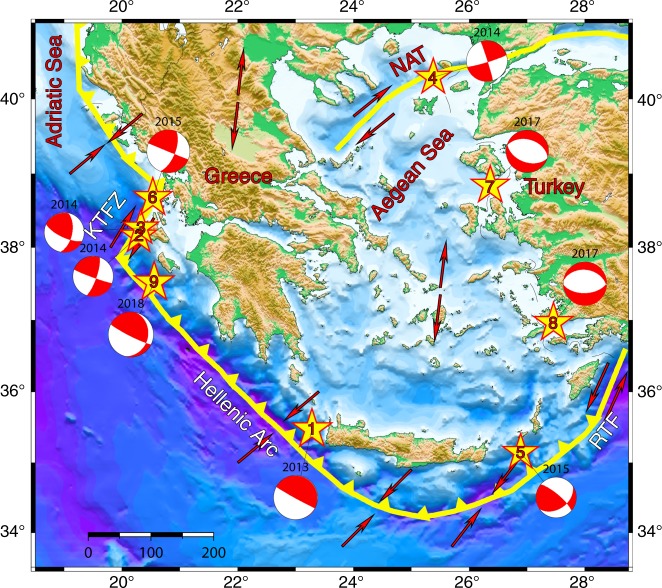
Epicentral distribution of considered mainshocks. The spatial distribution of the *M* ≥ 6.0 mainshocks that occurred in the Aegean area since October 2013 along with their fault plane solutions shown as lower hemisphere equal area projections. On the top of each ball the occurrence year is given, whereas the numbers inside the yellow stars representing the mainshock epicenters designate their sequential occurrence in time: (1) Crete, (2) Lixouri1, (3) Lixouri2, (4) North Aegean, (5) Karpathos, (6) Lefkada, (7) Lesvos, (8) Kos, (9) Zakynthos. The map is generated using the Generic Mapping Tool (http://www.soest.hawaii.edu/gmt;). Fault plane solutions were taken from http://Ideo.columbia.edu/gcmt

We apply the method to nine *M* ≥ 6 mainshocks (Table 1) occurred in the Aegean area in the last 5 years^25–27^ (see Fig. 1) including the *M* = 6.8 Zakynthos earthquake occurred on 25 October 2018, after the first manuscript submission. The study area is characterized by remarkably high seismic activity, with frequent *M* > 6 earthquakes that have caused severe casualties and damage during the last centuries, since historical information is available. Each mainshock was followed by a significant increase of the seismic rate in areas of tens of kilometers from the mainshock epicenter. The largest aftershocks are typically observed in the first part of the aftershock sequence and, in some cases, aftershocks as large as *M* ≥ 5 were recorded just a few hours after the mainshock.

**Table 1:** Information on the mainshocks with *M* > 6.0 that occurred in the Aegean area since 2013 and on the Ischia earthquake

Index	Code ame	Date	Occurrence time	Latitude	Longitude	Depth (km)	Loc. Magnitude	*N*1	*N*3	*δr*
1	Crete	12 Oct. 2013	13:11	35.471	23.281	47.0	6.7	5	7	91
2	Lixouri1	26 Jan. 2014	13:55	38.154	20.287	13.50	6.1	442	502	64
3	Lixouri2	3 Feb. 2014	03:08	38.266	20.323	9.40	6.0	288	367	57
4	North Aegean	24 May 2014	09:25	40.286	25.375	12.80	6.9	3	3	48
5	Karpathos	16 Apr. 2015	18:07	35.146	26.888	22.6	6.1	101	123	52
6	Lefkada	17 Nov. 2015	07:10	38.673	20.530	6.6	6.0	159	175	62
7	Lesvos	12 June 2017	12:28	38.839	26.362	11.80	6.1	116	126	45
8	Kos	20 July 2017	22:31	36.959	27.453	1.5	6.1	261	297	28
9	Zakynthos	25 Oct. 2018	22:54	37.341	20.5123	9.9	6.6	n.a.	n.a.	65
10	Ischia	21 Aug. 2017	18:57	40.738	13.897	1.24	3.9	3	3	0.9

We indicate with *N*_1_ and *N*_3_ the number of aftershocks with *M* > *M*_*m*_ − 3.0 recorded within 1 and 3 months, respectively, after the mainshock occurrence. *δr* is the distance in km between the mainshock epicenter and the recording station

For each mainshock we have investigated the ground velocity *V*(*t*) recorded at the closest seismic station during the first 3 days. In all cases we consider the vertical component of the seismogram but very similar results are found using the other two components. We identify the time *t*_max_ corresponding to the maximum peak ground velocity *V*_max_ and arbitrarily chose the origin time of the mainshock at the time *t*_0_ < *t*_max_ such that *V*(*t*_0_) = *qV*_max_. Different choices of *q* ∈ [0.25,0.75] do not alter our results. We then define the envelope function *μ*(*t*) as

2 $$\mu (t) = {\mathrm{log}}_{10}\left( {{\cal{H}}\left( {\hat V(t - t_0)} \right)} \right).$$

Here $\hat V(t - t_0)$ is the filtered signal, in the range [2,10] Hz, of the peak ground velocity *V*(*t*) shifted by *t*_0_ and ${\cal{H}}(x)$ is the Hilbert transformation. Finally, we obtain the average envelope $\bar \mu (t)$ via a smoothing procedure (see Methods section).

**Fig. 2:**
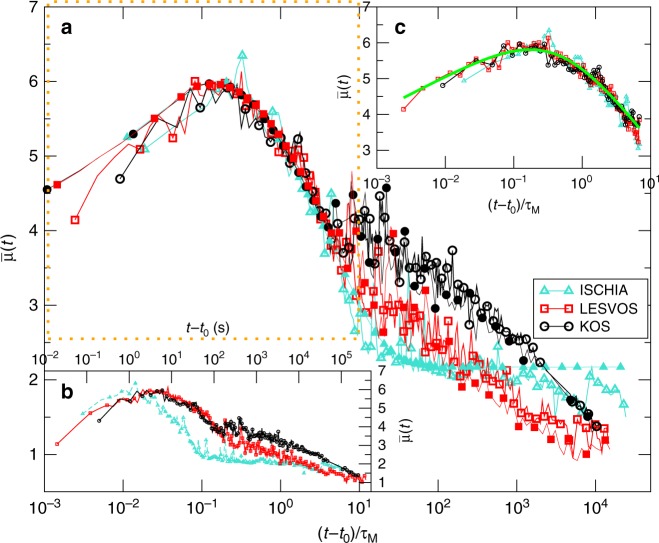
The average envelope function of the Kos, Lesvos, and Ischia earthquakes. **a** The average envelope function $\bar \mu (t)$ is plotted as a function of the time divided by the characteristic time *τ*_*M* _= 4.3 s for Ischia, *τ*_*M* _= 22.6 s for Kos, and *τ*_*M* _= 25.7 s for Lesvos. Different open symbols and colors are used for the three mainshocks (see legend). Filled symbols represent the theoretical envelope $\overline {\mu _{\mathrm{{th}}}} (t)$ obtained setting *b* = 1, *p* = 1.1 and implementing different choices of *K* and *c* for the three different mainshocks. **b** The same data of panel (**a**) without time rescaling. **c** A zoom inside the orange dotted rectangle of the main panel. The green line is the scaling function *F*(*x*) = 6 + log_10_(*x*) − 3.5log_10_(*x* + 0.43)

We plot in Fig. 2b the envelope $\bar \mu (t)$ of the two earthquakes (Lesvos and Kos) occurred in 2017 in the Aegean region. We include for comparison the *M* = 3.9 Ischia earthquake also occurred in 2017 in Italy. For this earthquake, because of technical problems with the seismic station, only the ground acceleration was available. A common feature of these three earthquakes is their location in or very close to three famous Mediterranean Isles during the summer touristic season and, consequently, their huge economic impact caused by the cancellation of many holiday bookings after their occurrence. In the main panel of Fig. 2 we plot the envelope $\bar \mu (t)$ rescaling the time scale by a fitting parameter *τ*_*M*_, different for each mainshock. We find that the three envelopes exhibit an excellent collapse up to the time (*t* − *t*_0_)/*τ*_*M*_ ≃ 10 (Fig. 2c), suggesting the same functional behavior

3 $$\bar \mu (t) = \mu _M + F\left( {(t - t_0)/\tau _M} \right).$$

**Fig. 3:**
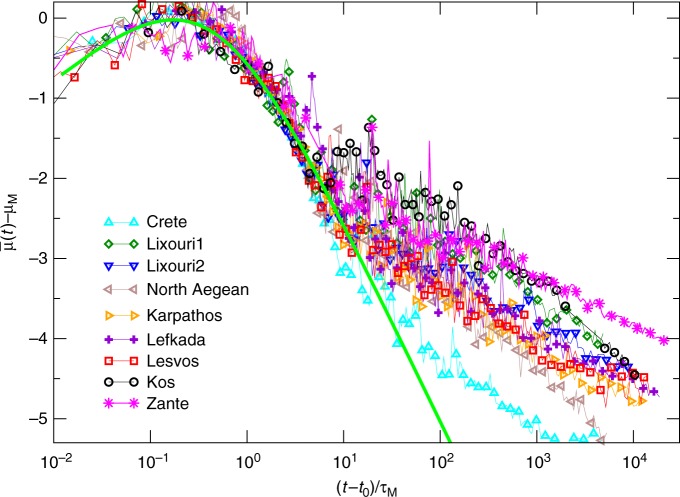
The average envelope function of the recent Aegean earthquakes. The quantity $\bar \mu _{\mathrm{{th}}}(t) - \mu _M$ is plotted versus (*t* − *t*_0_)/*τ*_*M*_ with: *τ*_*M* _= 16.5 s for Crete, *τ*_*M* _= 39.0 s for Lixouri1, *τ*_*M* _= 27.3 s for Lixouri2, *τ*_*M* _= 25.5 s for North Aegean, *τ*_*M* _= 12.9 s for Karpathos, *τ*_*M* _= 12.1 s for Lefkada, *τ*_*M* _= 25.7 s for Lesvos, *τ*_*M* _= 22.6 s for Kos, and *τ*_*M* _= 11.4 s for Zakynthos. The green continuous line is the scaling function *F*(*x*) = log_10_(*x*) − 3.5log_10_(*x* + 0.43)

The parameter *μ*_*M*_ in Eq. (3) represents the maximum value of $\bar \mu (t)$ and we define it the perceived magnitude since it provides the magnitude of ground shaking felt close to the station. We find that the three mainshocks present roughly the same value of *μ*_*M*_, a result expected for the Kos and Lesvos earthquakes with comparable magnitudes and similar epicentral distance from the recording station. In the case of the Ischia earthquake, this result can be primarily attributed to the smaller epicentral distance from the recording station (see Table 1) but also to soil conditions at the seismic station location^28^. Concerning the functional dependence of the scaling function *F*(*x*) in Eq. (3), the shape of the envelope function is dominated by different mechanisms such as source properties, attenuation, scattering effects, etc.^29^. The scaling collapse of Fig. 2c suggests that these features are, at a first approximation, incorporated in two seismic source-dependent parameters: the perceived magnitude *μ*_*M*_ and the horizontal time rescaling *τ*_*M*_. This result is confirmed by the scaling collapse also obtained for the other mainshocks, when plotting $\bar \mu (t) - \mu _M$ as a function of (*t* − *t*_0_)/*τ*_*M*_ (Fig. 3). In all cases we find that, for *x* < 10, *F*(*x*) ≃ log_10_(*x*) − *q*log_10_(*x* + *x*_0_) with *q* ≃ 3.5 (continuous curve in Fig. 2c) and *x*_0_ = (*q* − 1)*q*^*q*/(1−*q*)^ ≃ 0.43 such that the maximum value of *F*(*x*) is equal to zero. We assume the same functional dependence of *F*(*x*) for all mainshocks in order to reduce the number of fitting parameters in our method. This fit corresponds to a power law decay of the amplitude of coda-waves of *V*(*t*)^30^ which anticipates the exponential decay at longer timescales^29^. We wish to emphasize that our results are weakly affected by the specific choice of *F*(*x*) and we expect that other choices for *F*(*x*) lead to similar results.

**Fig. 4:**
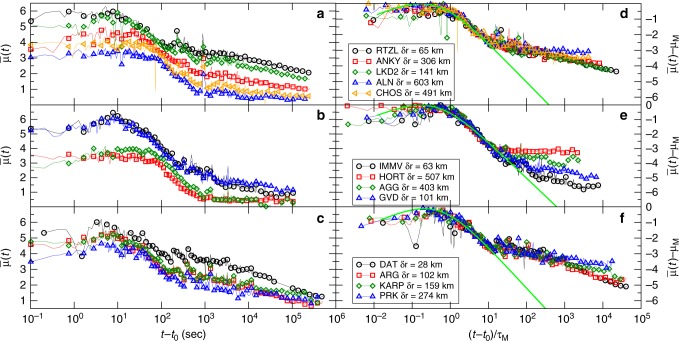
The average envelope function at different epicentral distances. **a**–**c** The average envelope function $\bar \mu (t)$ obtained from the signal recorded at seismic stations at different epicentral distances *δr* from the mainshock after the Zakynthos earthquake (**a**), Crete earthquake (**b**), and Kos earthquake (**c**). The station name and epicentral distances are reported in the figure legend. **d**–**f** The same quantity of the corresponding left panel plotted after the rescaling $\bar \mu (t)$ − *μ*_*M*_ and (*t* − *t*_0_)/*τ*_*M*_, with *τ*_*M* _= 11.4, 27.7, 16.1, 57.7, and 50.8 s for the stations *RTZL*, *ANKY*, *LKD*2, *ALN*, and *CHOS*, respectively, for the Zakynthos earthquake (**d**), *τ*_*M* _= 16.5, 57.7, 55.4, and 18.5 s for the stations *IMMV*, *HORT*, *AGG*, and *GVD*, respectively, for the Crete earthquake (**e**), and *τ*_*M* _= 22.6, 23.1, 25.6, and 41.8 s for the stations *DAT*, *ARG*, *KARP*, and *PRK*, respectively, for the KOS earthquake (**f**). The green continuous line is the scaling function *F*(*x*) = log_10_(*x*) − 3.5log_10_(*x* + 0.43)

Figure 4 shows $\bar \mu (t)$ for the same mainshock recorded at seismic stations at different epicentral distances *δr*. We found that, as expected, attenuation effects lead to a perceived magnitude *μ*_*M*_ which is a decreasing function of *δr*. At the same time, we observe that *τ*_*M*_ increases for increasing *δr* and this can be probably attributed to scattering effects. Nevertheless, the plot of $\bar \mu (t)$ − *μ*_*M*_ as a function of (*t* − *t*_0_)/*τ*_*M*_ (right panels of Fig. 4) still exhibits a good scaling collapse which is consistent with Eq. (3) for (*t* − *t*_0_)/*τ*_*M*_ < 10. The collapse extends up to (*t* − *t*_0_)/*τ*_*M*_ = *x*_B_ > 10, whereas for (*t* − *t*_0_)/*τ*_*M*_ > *x*_B_ the envelope presents an almost flat behavior $\bar \mu (t)$ = *μ*_B_ related to the contribution of background seismicity which is expected to be quite stationary in time. Figure 4 presents results for only three earthquakes: The more recent one (Zakynthos), the one with the largest $\bar \mu (t)$ − *μ*_*M*_ for *t* > *τ*_*M*_ (Kos) and the one with the smallest one (Crete). A similar behavior is observed in the other earthquakes.

### Theoretical interpretation

The main observation at the basis of our method concerns the clear separation of the envelope $\bar \mu (t)$ for the three mainshocks at times *t* > 10*τ*_*M*_ (Fig. 2), which we attribute to a different aftershock productivity, i.e. different *K* and *c* values. This temporal domain, as already observed in refs. ^15,31^, is indeed controlled by aftershock occurrence. If an aftershock with perceived magnitude *μ*_*i*_ has occurred at time *t*_*i*_, the envelope $\bar \mu (t)$ cannot become smaller than $\overline {\mu (t)} \simeq \mu _i$ at times *t* ≃ *t*_*i*_. Therefore, larger values of $\bar \mu (t)$, for equal *μ*_*M*_, indicate a higher occurrence rate of big aftershocks and, consequently, a greater aftershock productivity. In particular, the low level of *μ*(*t*) for *t* ∈ [50, 120] s in the case of the Ischia earthquake can be interpreted as a clear indication of a very small occurrence probability of *μ*_*i*_ ≥ 4 aftershocks in Ischia, in the hours following the mainshock. Indeed, only aftershocks with *μ*_*i*_ ≤ 3 have been subsequently recorded. Accordingly, from the low levels of $\bar \mu (t)$ recorded few minutes after the Ischia earthquake it would have been already possible to inform the authorities and issue a confirmatory announcement for mitigating the economic impact of the earthquake. The situation, conversely, was different after the Kos earthquake where $\bar \mu (t)$ has been stably above $\bar \mu (t) > 3.5$ in the first minutes and indeed tens of *μ*_*i*_ > 4 aftershocks occurred in the next 2 days (see Table 2). Similar indications can be obtained from the envelope function $\bar \mu (t)$ for all the *M* ≥ 6 mainshocks occurred in the Aegean area after 2013. Figure 3, indeed, confirms that dividing the time by the appropriate *τ*_*M*_, $\bar \mu (t) - \mu _M$ reveals a quite universal behavior up to *t*/*τ*_*M*_ < 10. For longer times the split of different envelopes is evident with important consequences for aftershock forecasting. In the following we present a forecasting tool which provides the quantitative estimation of the above considerations.

**Table 2:** The comparison between the observed *n*_obs_ and predicted *n*_pred_ (*T*) aftershocks

Code name	*n*_obs_	*n*_pred_ (20 min)	*n*_pred_ (1 h)	*n*_pred_ (2 h)
Crete	11	11	9	10
Lixouri1	455	157	390	396
Lixouri2	277	172	252	317
North Aegean	94	93	102	81
Karpathos	123	85	108	109
Lefkada	161	65	102	120
Lesvos	119	58	100	111
Kos	550	301	621	758
Zakynthos	480	291	472	515

We consider aftershocks with *μ*_*i*_ > *μ*_*M*_ − 3 in three learning periods *T* = 20 min, 1 h, and 2 h and for the testing period [*t*_in_, *t*_*f*_], with *t*_in _= 2 h and *t*_*f*_  = 72 h, except for Crete where *t*_*f*_  = 48 h and for Karpathos where *t*_*f*_  = 54 h. The quantity *n*_pred_(*T*) is obtained with the values of *K* and *c* listed in Table 3, expressing *t*_in_, *t*_*f*_, and *c* in seconds and using *b* = 1 and *p* = 1.1

### The forecasting method

Since aftershocks and mainshock mostly occur in the same tectonic environment, we expect that they present very similar attenuation and scattering properties. This is confirmed by the envelope function of the largest aftershocks, with occurrence time *t*_*i*_ and perceived magnitude *μ*_*i*_, which is well described by $\bar \mu _i(t - t_i) = \mu _i + F\left( {(t - t_i)/\tau _M} \right)$ with the same scaling function *F*(*x*) and the same *τ*_*M*_ of the mainshock. Accordingly, given a mainshock with perceived magnitude *μ*_0_ = *μ*_*M*_ occurred at time *t*_0_ = 0 and an aftershock sequence {*t*_*i*_, *μ*_*i*_} (*i* ≥ 1) we expect that the envelope function is given by

4 $$\bar \mu _{\mathrm{{th}}}(t) = \mathop {{\max }}\limits_{i:t_i < t} \bar \mu _i(t - t_i) + \mu _{\mathrm{B}},$$

where the maximum must be evaluated over all events, including the mainshock, occurred at times *t*_*i*_ < *t*, and *μ*_B_ corresponds to the background level of ground velocity during stationary periods. Equation (4) just states that $\bar \mu _{\mathrm{{th}}}(t)$ initially follows the mainshock envelope but as soon as a sufficiently strong aftershock occurs, $\bar \mu _{\mathrm{{th}}}(t)$ increases up to $\bar \mu _{\mathrm{{th}}}(t) = \mu _i$ and decays according to *F*((*t* − *t*_*i*_)/*τ*_*M*_) unless an other aftershock occurs and the signal can increase again. Equation (4) is supported by the scaling collapse (Fig. 4) of the envelope of the signal recorded at different stations which indicates that, even for distant stations, $\bar \mu (t)$ is dominated by the aftershock occurrence up to the time *t*_B_. Our method is based on the idea to infer the statistical features of aftershock occurrence from $\bar \mu (t)$ in the time interval [*τ*_*M*_, *t*_B_]. In order to reduce the contamination of background seismicity, in the following we restrict to the signal recorded at the closest station.

Figure 2 shows that it is possible to generate a synthetic aftershock sequence $\{ t_i,\mu _i\}$, for each one of the three mainshocks, which presents a $\bar \mu _{\mathrm{{th}}}(t)$ such that $\bar \mu _{{\mathrm{{th}}}}(t) \simeq \bar \mu (t)$ at different times $t$. More specifically, the aftershock sequence is simulated assuming that aftershock occurrence follows a generalized Poisson process with occurrence rate $\lambda (t)$ given by the OU law (Eq. 1) (see Methods section). The agreement between the theoretical $\bar \mu _{\mathrm{{th}}}(t)$ and the instrumental $\bar \mu (t)$ represents the key ingredient of our method. Indeed, since $\bar \mu _{\mathrm{{th}}}(t)$ strongly depends on $\lambda (t)$, we observe that different choices of the parameters (*b*, *p*, *c*, *K*) in the synthetic sequence lead to a very different $\bar \mu _{{\mathrm{{th}}}}(t)$. On the other hand, different synthetic sequences implementing the same value of the parameters (*b*, *p*, *c*, *K*), correspond to a similar average envelope $\bar \mu _{{\mathrm{{th}}}}(t)$. As a consequence, the values of the model parameters (*b*, *p*, *c*, *K*), which represent the best description of the aftershock decay rate, are expected to correspond to those values which provide the best agreement between $\bar \mu _{\mathrm{{th}}}(t)$ and $\bar \mu (t)$.

The possibility to extrapolate the parameters (*b*, *p*, *c*, *K*) from the decay of the envelope function has been already recognized in Lippiello et al.^15^. The central assumption was that $\bar \mu (t)$, for times *t* − *t*_0_ > 10*τ*_*M*_, obeys a logarithmic decay, $\bar \mu (t)$ = *μ*_*M*_ − Δ*μ* − *ϕ* log (*t*) and that the parameters (Δ*μ*, *ϕ*) are somehow related to *K* and *c*. The novel and more efficient method developed and applied in this study does not rely on assumptions on the functional form of $\bar \mu (t)$. In particular, it can be implemented in an automatic and almost real-time procedure which provides the occurrence probability of strong ground shaking in a few minutes after the mainshock. The procedure is detailed in the Methods section and here we outline the main steps. In the first step we obtain the best fit of *μ*_*M*_ and *τ*_*M*_ considering only data for *t* ≤ 100 s. In the second step we consider the learning period *t* ∈ (0, *T*) and, for a given set of parameters *K*, *c*, *b*, *p*, we simulate an aftershock sequence {*t*_*i*_, *μ*_*i*_} assuming that the aftershock occurrence probability in time is given by Eq. (1) with Δ*M*  = *μ*_*M*_ − *μ*_*i*_. We then assume the Ishimoto and Iida law^24^ for the peak ground velocity which corresponds to an exponential law for the frequency distribution of the aftershock perceived magnitude $P(\mu _i) \propto 10^{ - \beta \mu _i}$. This is related^23^ to the Gutenberg–Richter law for the frequency distribution of earthquake magnitude and, in order to reduce the number of fitting parameters, we always consider *β* = *b* = 1 which is a quite realistic value for all the considered mainshocks. For the same reason we also consider a fixed *p* value, *p* = 1.1. We have explicitly verified that routines with variable *b* and *p* values do not produce any improvement.

The subsequent step is the evaluation of $\bar \mu _{\mathrm{{th}}}(t)$ along with the root mean square deviation $\chi (T) = {\int}_0^T {\mathrm{d}} t\left( {\bar \mu (t) - \bar \mu _{\mathrm{{th}}}(t)} \right)^2$ between the instrumental and theoretical envelope functions. By means of a simulated annealing procedure (see Methods section) we find the best parameter values which correspond to the minimum value of *χ*(*T*). This is a fast procedure, of the order of few minutes on standard CPU, which can be repeated as soon as time goes on and new data of $\bar \mu (t)$ become available. In this way we can obtain the best set of parameters in the OU law at different times *T* after the mainshock.

### Testing procedure

**Fig. 5:**
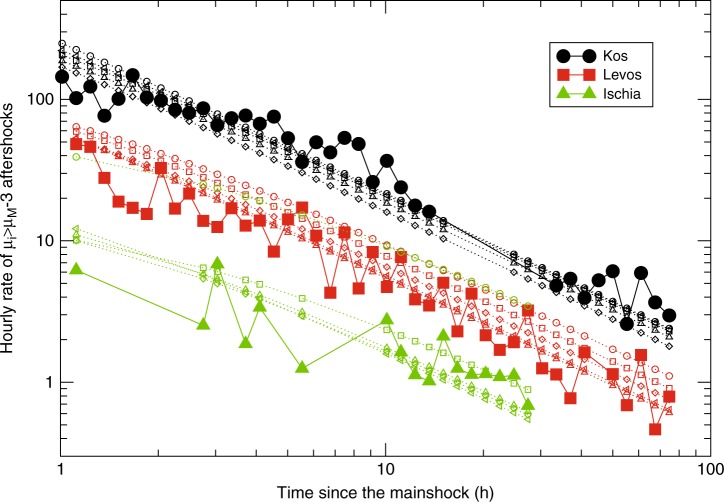
Test of the method for the Kos, Lesvos, and Ischia earthquakes. We use filled large symbols for the hourly rate *λ*(*t*) of aftershocks with *μ*_*i*_ > *μ*_*M*_ − 3 with different symbols and colors for different mainshocks (see legend). Small empty symbols are used for the predicted aftershock rate obtained using in the OU law Eq. (1) the values of *K* and *c* provided by our model. Different colors correspond to different mainshocks whereas different symbols (circles, squares, diamonds, triangles up, triangles left) correspond to different learning periods (*T* = 1/6, 1/4, 1/2, 1, and 6 h), respectively

To test the method efficiency, the parameters *K*, *c*, and *μ*_B_ obtained as best fit in the learning period (0, *T*) are used to forecast the occurrence of aftershocks with perceived magnitude *μ*_*i*_ > *μ*_*M*_ − 3 in the testing period [*t*_in_ = 2 h, *t*_*f*_ = 72 h]. The choice *μ*_*i*_ > *μ*_*M*_ − 3 represents a compromise between a sufficient number of aftershocks for robust statistical tests and considerable ground shaking induced by each aftershock. Aftershocks are identified according to the procedure outlined by Peng et al.^8^ selecting among all peaks of the envelope *μ*(*t*) with *μ*_peak_ ≥ *μ*_*M*_ − 3. This procedure allows us to identify many more aftershocks than those reported in the official Greek catalog. Figure 5 presents with filled symbols the hourly rate *λ*(*t*) of *μ*_*i*_ > *μ*_*M*_ − 3 aftershocks identified starting from 1 h after the mainshock. Different colors are used for the different mainshocks. The Kos sequence produces roughly 30 times more aftershocks than the Ischia one. In Kos and Lesvos sequences, *λ*(*t*) is consistent with the OU law with *p* ≃ 1, whereas the Ischia sequence comprises only a few aftershocks and the OU decay is not observed. In the same figure open symbols are used for the hourly rate of events with *μ*_*i*_ > *μ*_*M*_ − 3 predicted by implementing in the OU law (Eq. (1)) the best-fit parameters *K*, *c* obtained in the learning period (0, *T*). Different colors correspond to different mainshock sequences whereas different symbols, for each sequence, correspond to different values of *T*. Results show that for the Kos and Lesvos mainshocks an accurate prediction of subsequent aftershock occurrence could be available only ten minutes after the mainshock. In the Ischia earthquake, the forecasting after 10 min is not so efficient but for *T* ≥ 15 min the agreement between predicted and recorded *λ*(*t*) is satisfactory.

**Fig. 6:**
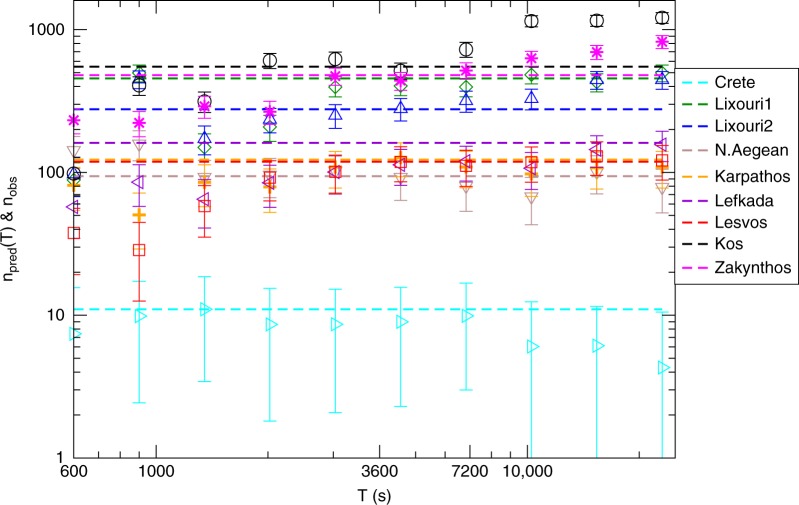
Short-term test of the method for the recent Aegean earthquakes. We compare the number of observed and predicted aftershocks with *μ*_*i*_ > *μ*_*M*_ − 3 in the testing period [*t*_in_, *t*_*f*_], with *t*_in _=2 h and *t*_*f* _= 72 h except for Crete where *t*_*f* _= 48 h and for Karpathos where *t*_*f* _= 54 h. Open symbols represent the value of *n*_pred_(*T*) for different values of the testing period *T*. The error bars correspond to statistical fluctuations in the number of expected aftershocks for fixed *K* and *c* values. Dashed horizontal lines represent n_obs_, i.e. the number of identified aftershocks according to the method described in ref. ^8^. Different symbols and colors are used for different mainshocks (see legend)

In order to better verify the efficiency of our method, we apply the same procedure to estimate the best OU parameters *K* and *c* for all the *M* ≥ 6 mainshocks occurred after 2013 in the Aegean area (see Table 1). The best values of *K* and *c* obtained by our procedure for different learning periods *T* and different mainshocks are listed in Table 3. In Fig. 6 we plot with continuous lines the number of aftershocks *n*_obs_ with perceived magnitude *μ*_*i*_ > *μ*_*M*_ − 3 identified in the testing interval [2, 72 h] after each mainshock. Results show huge differences (up to 6000%) in the number of observed aftershocks, among different sequences, even for similar magnitude mainshocks. The number of identified aftershocks is compared with the number of *μ*_*i*_ > *μ*_*M*_ − 3 aftershocks predicted by our model *n*_pred_(*T*) which, according to Eq. (1), is given by $n_{\mathrm{{pred}}}(T) = \frac{{K10^{b{\mathrm{\Delta }}M}}}{{p - 1}}\left( {\left( {t_{\mathrm{{in}}} + c} \right)^{1 - p} - \left( {t_f + c} \right)^{1 - p}} \right)$, with Δ*M* = 3, *b* = 1 and *p* = 1.1 fixed. We use *t*_in_ = 2 h and *t*_*f*_ = 72 h and plot results for different testing periods *T* with open symbols in Fig. 6. Results have been also reported in Table 2 and a zoom can be found in Supplementary Fig. 1. For all mainshocks we found out that our procedure efficiently forecasts the number of observed aftershocks with a discrepancy typically smaller than 20%. Results also show that the best agreement is obtained for a learning period *T* ∈ (0.5 h, 2 h). The agreement tends to become worse for increasing *T* probably because of the contribution of higher order generation aftershocks, not considered in our study. Indeed, the possibility of each aftershock to trigger its own aftershocks leads to deviations from the pure OU law and the estimate of parameters (*K*, *c*) according to Eq. (1) becomes less and less accurate for increasing time^32–34^. Notice that for testing periods with *T* > 2 h, the testing and learning periods overlap. We make this choice in order to have a single value of *n*_obs_ in Fig. 6 and in Table 2 and this simplifies the presentation. At the same time the overlap is null in the more relevant temporal window *T* ≤ 2 h where our method produces the best agreement. Similar results are also found for a testing period with *t*_in_ = 1 h, as illustrated in the Supplementary Figs. 2 and 3 and in the Supplementary Table 1.

**Table 3:** The estimated parameters *K* and *c*

Code name	*K* (*T* = 20 min)	*c* (*T* =20 min)	*K* (*T*= 1 h)	*c* (*T* = 1 h)	*K* (*T* = 2 h)	*c* (*T* = 2 h)
Crete	0.0100	16	0.0084	3.75	0.0092	1.51
Lixouri1	0.1286	285	0.3174	159	0.3227	215
Lixouri2	0.1397	80	0.2045	155	0.2579	156
North Aegean	0.0757	281	0.0834	198	0.0663	218
Karpathos	0.0740	163	0.0952	283	0.0956	261
Lefkada	0.0532	282	0.0830	238	0.0976	153
Lesvos	0.0470	205	0.0824	365	0.0907	296
Kos	0.2434	53	0.5020	38	0.6132	52
Zakynthos	0.2375	265	0.3850	240	0.4185	165

For each mainshock we consider three different learning periods *T* = 20 min, 1 h, 2 h. The time *c* is expressed in seconds and the parameter *K* is in units of seconds^*p*−1^

**Fig. 7:**
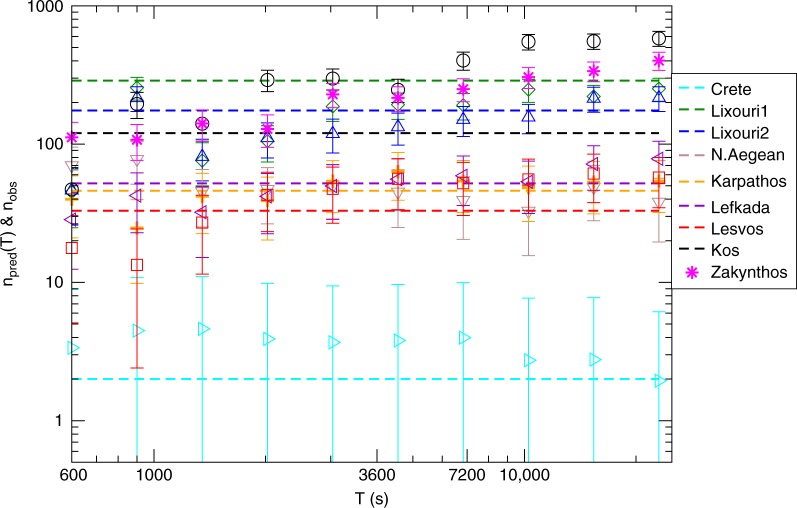
Long-term test of the method for the recent Aegean earthquakes. The same plot as in Fig. 6 for the number of predicted aftershocks *n*_pred_(*T*) (open symbols) with *μ*_*i*_ > *μ*_*M*_ − 3 in the testing period [7, 90] days. Dashed horizontal lines is the number of aftershocks with *m*_*i*_ > *M*_*m*_ − 3 recorded in the Greek catalog in the interval [7, 90] days. Aftershocks are defined as events occurring within a radius of size $L_M = 0.02 \times 10^{0.5m_M}$ from each mainshock epicenter

The same comparison between observed and predicted aftershock number is performed in Fig. 7. In this case we plot the number *n*_pred_(*T*) of *μ*_*i*_ > *μ*_*M*_ − 3 aftershocks predicted by our model in the temporal range [*t*_in_ = 7, *t*_*f*_ = 90] days when short-term catalog incompleteness is not relevant. We therefore directly compare with the number *n*_aft_ of *m*_*i*_ > *M*_*m*_ − 3 aftershocks included in the Greek earthquake catalog in the same temporal period. We still found a good agreement between prediction and observation, which shows that in only a few minutes after the mainshock it is already feasible to provide insights in the evolution of seismicity in the following months. There is an exception related to the 2014 North Aegean sequence since the model predicts about 70 aftershocks with *m*_*i*_ > *M*_*m*_ − 3 in the interval [7, 90] days whereas no aftershock is included in the Greek catalog. In this case we observe an abrupt change of the seismic rate, in the mainshock area, which is not consistent with the OU law and, therefore, it is not predicted by our procedure. On the other hand, in the case of the Zakynthos earthquake we find *n*_pred_(*T* = 1 h) = 220 ± 30 and we cannot compare with *n*_aft_ in the Greek catalog, since the testing period is not yet finished. The same analysis of Fig. 7 for a testing period of [30, 90] days is presented in Supplementary Fig. 4.

## Discussion

Our results have shown that, in only 30 min after a mainshock, it is possible to have an accurate forecasting of the aftershock occurrence rate in the subsequent days. We wish to emphasize that, to our knowledge, existing methods are not capable to provide such an efficient forecasting in a very short learning period of *T* ~ 1 h, like the one here considered. The most refined way to take explicitly into account aftershock incompleteness is the Omi et al. method^11,16,20^. This method is based on a Bayesian estimate of the completeness magnitude which, however, requires a sufficient number of identified aftershocks. In Omi et al. (2018) the method has been applied only to sequences with at least 30 events in the learning period. In the case of the mainshocks considered in our study, the number of identified aftershocks, at temporal distances smaller than *T* ~ 2 h reported in the Greek earthquake catalog, is typically smaller than 20 and this makes the prediction according to the Omi et al. method very unstable.

In conclusion, we have presented a novel method for aftershock forecasting where the parameters of the OU law are extracted from the ground velocity recorded some minutes after the mainshock. The accuracy of the method has been verified by means of retrospective tests on recent Greek mainshocks and future developments correspond to the validation by means of prospective tests. Refinements of the method can be obtained by incorporating higher order generation aftershocks which should contribute to improve the agreement for learning periods larger than 1h.

## Methods

### The average envelope

The quantity $\bar \mu (t)$ is obtained by means of a logarithmic smoothing procedure that corresponds to the average value of *μ*(*t*) over windows of increasing duration Δ*t*_*k*_ = Δ*t*_0_(1 + *ζ*)^*k*^ with Δ*t*_0_ = 0.1 s and *ζ* = 0.005.

### Algorithm to invert the OU parameters

The algorithm is composed of four separate routines.

The first routine considers $\bar \mu (t)$ restricted to the time interval [*t*_0_, *t*_0_ + 100 s] and looks for the parameters *μ*_*M*_ and *τ*_*M*_ which minimize the quantity

$$\chi = \left( {\bar \mu (t) - \mu _M - {\mathrm{log}}_{10}((t - t_0)/\tau _M) + q{\mathrm{log}}_{10}((t + \tau _0)/\tau _m + x_0)} \right)^2$$

with *q* = 3.5.

The second routine generates, for a given set of parameters (*b*, *p*, *c*, *K*), an aftershock sequence. More precisely, we assume that the number of aftershocks follows a Poisson distribution with average value *K*10^*b*Δ*M*^ with Δ*M* = 3. We randomly extract the occurrence time of each aftershock *t*_*i*_ from the probability distribution $P(t_i) = \frac{{p - 1}}{{c^{1 - p}}}(t_i + c)^{ - p}$. To each aftershock we associate a perceived magnitude *μ*_*i*_ = *μ*_*M*_ − Δ*M* + *m*_*i*_ with *m*_*i*_ is extracted from the GR law $P(m_i) = b{\mathrm{log}}(10)10^{ - bm_i}$, without any extra constraint, i.e. *μ*_*i*_ can be greater than *μ*_*M*_.

The third routine uses the information on *τ*_*M*_ and *μ*_*M*_, provided by the first routine, and starting from an aftershock sequence {*t*_*i*_, *μ*_*i*_} generated by the second routine, gives in output $\bar \mu _{\mathrm{{th}}}(t)$ according to Eq. (4). More precisely, the time is discretized in steps of 0.05 s and at each time *t*, we evaluate the quantity $\bar \mu _i(t - t_i) = \mu _i + {\mathrm{log}}_{10}\left( {(t - t_i)/\tau _M} \right) - 3.5{\mathrm{log}}_{10}\left( {(t - t_i)/\tau _M + x_0} \right)$, for all aftershocks with *t*_*i*_ < *t*. The quantity *μ*_th_(*t*) is given by the maximum value of $\bar \mu _i(t - t_i)$ and we use the same logarithmic smoothing applied to the instrumental envelope to obtain $\bar \mu _{\mathrm{{th}}}(t)$.

The fourth routines finds the best OU parameters (*b*, *p*, *c*, *K*) via the minimization of the discrepancy between the theoretical and the instrumental envelope, up to a learning time *T*. This is achieved by means of the algorithm developed by Bottiglieri et al.^35^ for the log-likelihood maximization. More precisely, for a given parameter set (*b*, *p*, *c*, *K*), we use *n*_real_ times the third routine to obtain *n*_real_ independent realizations of the envelope $\bar \mu _{\mathrm{{th}}}^{(j)}(t)$ with *j* = 1,..., *n*_real_. For each realization we evaluate the quantity $\chi ^{(j)}(T) = {\int}_0^T {\mathrm{d}} t\left( {\bar \mu (t) - \bar \mu _{\mathrm{{th}}}^{(j)}(t)} \right)^2$ and then define *χ*(*T*) as the minimum value of *χ*^(*j*)^(*T*) for *j* = 1, ..., *n*_real_. The parameters (*b*, *p*, *c*, *K*) are then updated according to the algorithm of Bottiglieri et al.^35^ until we find the best values which correspond to the minimum of *χ*(*T*). To reduce the number of parameters we fix *b* = 1 and *p* = 1.1. The code is available from the authors upon request.

In the limit *n*_real_ → ∞ the procedure identifies the synthetic aftershock sequence which, according to Eq. (4), gives the best description of the instrumental signal and therefore the corresponding (*b*, *p*, *c*, *K*) is considered the best parameter set. We have verified (see Supplementary Fig. 5) that the procedure appears quite stable for *n*_real_ ≥ 10. In order to reduce the computational time, results presented in this manuscript are obtained for *n*_real_ = 10. We have also explored two alternative definitions of *χ*(*T*): The average value of *χ*^(*j*)^(*T*) for *j* = 1, ..., *n*_real_ and its median value. We have found that the three different definitions lead, for *n*_real_ ≥ 10, to a similar number of predicted aftershocks *n*_pred_(*T*) within an uncertainty typically smaller than the 20%.

## Supplementary information

Peer Review File

Supplementary Information

## Data Availability

All relevant data are available from the corresponding author upon reasonable request.
