# Supplementary material for: Forecasting of the first hour aftershocks by means of the perceived magnitude
Source: Nat Commun. 2019 Jul 4;10:2953. doi: 10.1038/s41467-019-10763-3 (PMC6609666; doi:10.1038/s41467-019-10763-3)
Supplement: Supplementary file 2 — Supplementary Information [file 41467_2019_10763_MOESM2_ESM.pdf]

Reviewers' comments:

Reviewer #1 (Remarks to the Author):

I have carefully read and reviewed the manuscript entitled “Forecasting of the first hour aftershocks by means of the perceived magnitude” by E. Lippiello et al. The manuscript proposed a method to estimate the parameters of the modified Omori and the Gutenberg-Richter laws from a waveform data and to forecast the aftershock numbers from the first hour data after a main shock. Given the importance of the rapid evaluation of earthquake risks after a damaging earthquake, this contribution is very important in the field, and this manuscript would merit publication in Nature Communications. The followings are my minor comments.

- Please clearly describe the relationship of the current manuscript to your previous 2016 GRL paper. These two works look similar at first glance, although I believe that the current manuscript gives more promising results.

- The authors assumed the exponential distribution for the frequency of the perceived magnitude. This relation is known as the Ishimoto-Iida law. Please see the below references.

Ishimoto, M., & Iida, K. Observations sur les séismes enregistrés par le micro-séismographe construit dernièrement (1), Bull. Earthq. Res. Inst., Univ. Tokyo 17, 443–478 (1939; in Japanese with French abstract).

Kato, M. Revisiting the Ishimoto–Iida Law for Strong-Motion Seismograms: A Case Study at CEORKA Network, Japan. Bulletin of the Seismological Society of America 104, 497–502 (2013).

- If I understand the manuscript correctly, the authors calculated the theoretical envelope function $\mu_{th}(t)$ from a single synthetic aftershock sequence. However, different realizations(simulations) of synthetic sequences result in different envelope functions. Therefore, it would be natural for me to define $\mu_{th}(t)$ as the envelope function averaged over many synthetic sequences.

- Typos in references

Please change ref. 9 to

Omi, T., Ogata, Y., Hirata, Y. & Aihara, K. Forecasting large aftershocks within one day after the main shock. Sci. Rep. 3, 2218 (2013).

Please change ref. 13 to

Omi, T., Ogata, Y., Shiomi, K., Enescu, B., Sawazaki, K. & Aihara, K. Automatic aftershock forecasting: A test using real-time seismicity data in Japan, Bull. Seismol. Soc. Am. 106, 2450 (2016).

Please change ref. 23 to

Sawazaki, K. & Enescu, B. Imaging the high-frequency energy radiation process of a main shock and its early aftershock sequence: The case of the 2008 Iwate-Miyagi Nairiku earthquake, Japan, J. Geophys. Res. Solid Earth 119, 4729–4746 (2014).

Reviewer #2 (Remarks to the Author):

This paper presents the interesting idea of using the envelope of the velocity seismogram to estimate the aftershock rate soon after a mainshock, even while the coda waves are still present in the seismogram and traditional earthquake catalogs are incomplete.

The paper demonstrates a collapse of the early-time velocity envelopes for 8 earthquakes, after the records are scaled, shifted, and normalized. The non-collapsing part of the envelope is taken to reflect differences in early aftershock activity. The parameters of the Omori's law are estimated by simulating aftershock sequences, computing a theoretical envelope for each simulated aftershock sequence, and then tuning the simulations to match the observed envelope. The authors then demonstrate how the tuned Omori's law predicts later aftershock activity measured either by counting high-frequency spikes or by using the actual regional earthquake catalog.

The idea is very promising but I was left confused by a number of issues, and was unable to reproduce a key figure with the data reported in the manuscript.

1) The inversion for the Omori parameters is inadequately described. A simulated aftershock sequence is generated from Omori's law, presumably with magnitudes randomly assigned from the Gutenberg-Richter distribution, and perceived magnitudes assigned based on the relative magnitude between the simulated aftershock and the mainshock. The envelope is computed, and then tuned by iterating on the Omori parameters. So is a single new simulated catalog generated for each update to the Omori parameters? If so, this seems somewhat unstable. I don't see how we could possibly be sampling all of the expected variability in the magnitudes of the aftershocks. Would it not be better to use some kind of average or median envelope based on a multitude of simulations for each parameter iteration? Are aftershocks allowed to be larger than the mainshock? I think you need to explain how magnitudes are assigned, and demonstrate that your method converges to the same parameters as would be obtained if you generated say, 100 simulations per parameter update and took the average or median theoretical envelope.

2) I was unable to reproduce a key figure. Using the parameter values in Table S2 I was unable to reproduce the predictions in Table S1 and figure 5. I came to my predicted numbers by integrating the Omori-Utsu law (Eq. 1) over the specified test interval/forecast window. I note that there are some entries in Table S1 that seem likely to be typos, and there is some confusion in the table caption. I'm quite confident that you mean a test period of [1h, 3days] and a learning period of [20min,1h,2h], but then your longest learning period overlaps with your test period. Was this intentional? I get forecast numbers that are roughly twice those reported using the specified parameters. Anwyay, the claim in the abstract of the forecast always being with 20% seems erroneous, given a quick glance at the numbers in Table S1. Is this value meant to apply only to a certain learning and test interval?

The authors should comfirm their calculations and check that all of the Omori parameters are correctly reported in Tables S1 and S2, as well as elsewhere in the manuscript (e.g. $b=1$, $p=1.1$).

3) It seems this paper could benefit from a more comprehensive analysis, using more data. Why only look at the closest station? The closest station is likely to be clipped, and additional stations would bring in additional data, allowing you to assess the precision and robustness of your method on a distribution of station distances for a single event. This seems all the more important given that you are using only 8 area earthquakes. Why only look at 8 regional earthquakes in the first place? You are making the case that you have discovered somethIng fundamentally new and useful, but only demonstrating it with a bare handful of earthquakes.

4) You observe that the best agreement is obtained for a learning period less than 2h. You

attribute this to secondary triggering. I find this very confusing. Your forecast should get worse with time away from the learning period due to secondary triggering, but it makes no sense that extending the learning period should degrade the forecast, precisely because the longer learning period captures more of the secondary triggering. I also do not buy the explanation that the large misfit for the N. Aegean sequence can be explained by the change in slope. The slope change is *very* subtle, and the same change in slope is present in the Kos data.

5) Presentation: There are a number of typos in this mansucript, including in the figure legends. Many of the figures are hard to read. For example, it is very difficult to tell open and filled symbols (data and model) apart in figure 2. I also find it very difficult to see all of the symbols in Figure 5.

We thank both reviewers for the careful reading of our manuscript and for the very interesting comments and remarks. We have properly taken them into account in the revised version of the manuscript. In the following you can find a detailed answer to each specific remark.

"Answer to Reviewer #1"

We thank the reviewer for considering our contribution "very important in the field" and for considering that our "manuscript would merit publication in Nature Communications". In the following we answer to each comment.

A) The reviewer writes:" Please clearly describe the relationship of the current manuscript to your previous 2016 GRL paper. These two works look similar at first glance, although I believe that the current manuscript gives more promising results."

We take into account this reviewer's observation in the Section "The forecasting method" of the revised version of the manuscript where we better discuss the relationship between the two manuscripts.

B) The reviewer writes:"The authors assumed the exponential distribution for the frequency of the perceived magnitude. This relation is known as the Ishimoto-Iida law. Please see the below references. Ishimoto, M., & Iida, K. Observations sur les sismes enregistrs par le micro-sismographe construit dernirement (1), Bull. Earthq. Res. Inst., Univ. Tokyo 17, 443478 (1939; in Japanese with French abstract). Kato, M. Revisiting the IshimotoIida Law for StrongMotion Seismograms: A Case Study at CEORKA Network, Japan. Bulletin of the Seismological Society of America 104, 497502 (2013)."

We thank the reviewer for drawing our attention to these very interesting papers. Indeed we were unaware of the Ishimoto-Ida (II) empirical law, confirming the observation in Kato (2013) that the II law has received less attention than the GR law. In the revised version of the manuscript we refer to the II law and cite the two suggested papers. In particular we refer to Kato (2013) to stress that working directly with ground shaking "we do not have to rely on assumptions on seismicity."

C) The reviewer writes:" If I understand the manuscript correctly, the authors calculated the theoretical envelope function $\mu_{th}(t)$ from a single synthetic aftershock sequence. However, different realizations(simulations) of synthetic sequences result in different envelope functions. Therefore, it would be natural for me to define $\mu_{th}(t)$ as the envelope function averaged over many synthetic sequences."

We thank the reviewer for this suggestion also given by the reviewer 2 (Answer 1c). We consider, for given values of K and c, n_{real} independent realizations of the synthetic catalog and we indicate with $\overline{\mu}_{th}^{(j)}(t)$ the theoretical envelope given by Eq.(4) for the j-th realization. We then define $\chi^{(j)}(T) =$

$\int_0^T dt \left(\overline{\mu}(t) - \overline{\mu}_{th}^{(j)}(t)\right)^2$ the root mean square deviation for the j-th realization. The best set of parameter K, c is the one that minimizes the quantity $\chi(T)$ and we adopt three different definitions of $\chi(T)$: i) the average value of $\chi^{(j)}(T)$, ii) the median value of $\chi^{(j)}(T)$, iii) the minimum value of $\chi^{(j)}(T)$, for $j = 1, .., n_{real}$. We obtain for the three definitions almost stable results for $n_{real} \geq 10$ (see Answer 1e to reviewer 2). In the revised version we adopt the definition (iii) and explicitly discuss this choice in the Methods section.

D) We have corrected all indicated typos in the references.

"Answer to Reviewer #2"

We thank the reviewer for judging our idea "interesting" and "very promising". In the following we answer to each remark:

1a) The reviewer writes: " The inversion for the Omori parameters is inadequately described."

After a careful reading back of the manuscript we must agree with the reviewer that the inversion procedure was cryptic. In the revised version of the manuscript we take advantage of the Methods section to provide a detailed description of the procedure adopted to invert the Omori parameters.

1b) The reviewer writes: "A simulated aftershock sequence is generated from Omori's law, presumably with magnitudes randomly assigned from the Gutenberg-Richter distribution, and perceived magnitudes assigned based on the relative magnitude between the simulated aftershock and the mainshock. The envelope is computed, and then tuned by iterating on the Omori parameters."

The reviewer correctly understands the adopted procedure which is now clearly described in the Methods section.

1c) The reviewer writes: "So is a single new simulated catalog generated for each update to the Omori parameters? If so, this seems somewhat unstable. I don't see how we could possibly be sampling all of the expected variability in the magnitudes of the aftershocks. Would it not be better to use some kind of average or median envelope based on a multitude of simulations for each parameter iteration?"

We thank the reviewer for this suggestion also given by the reviewer 1 (Answer C). We consider, for given values of K and c, n_{real} independent realizations of the synthetic catalog and we indicate with $\overline{\mu}_{th}^{(j)}(t)$ the theoretical envelope given by Eq.(4) for the j-th realization. We then define $\chi^{(j)}(T) = \int_0^T dt \left(\overline{\mu}(t) - \overline{\mu}_{th}^{(j)}(t)\right)^2$ the root mean square deviation for the j-th realization. The best set of parameter K, c is the one that minimizes the quantity $\chi(T)$ and

we adopt three different definitions of $\chi(T)$: i) the average value of $\chi^{(j)}(T)$, ii) the median value of $\chi^{(j)}(T)$, iii) the minimum value of $\chi^{(j)}(T)$, for $j = 1, .., n_{real}$. We obtain for the three definitions almost stable results for $n_{real} \geq 10$ (see Answer 1e to reviewer 2). In the revised version we adopt the definition (iii) and explicitly discuss this choice in the Methods Section.

1d) The reviewer writes: "Are aftershocks allowed to be larger than the mainshock?"

We extract aftershocks from a Gutenberg-Richter probability distribution without any constraint on the upper aftershock magnitude: An aftershock, in principle, can be larger than the mainshock. However, according to the Omori best parameters, obtained with our inversion procedure, the scenario of an aftershock larger than the mainshock is very unlikely to occur. Indeed, an aftershock larger than the mainshock produces a very large peak in the simulated envelope and this causes very large $\chi(T)$ values. This situation, even possible, is therefore 'avoided' by the $\chi(T)$ minimization procedure. We clarify how magnitude are assigned in the Method section of the revised manuscript.

1e) The reviewer writes: "I think you need to explain how magnitudes are assigned, and demonstrate that your method converges to the same parameters as would be obtained if you generated say, 100 simulations per parameter update and took the average or median theoretical envelope."

In order to follow the referee suggestion we have verified the convergence of our procedure for an increasing number n_{real} of independent realizations, for the three different definitions of $\chi(T)$ (answer 1c). We find that, for the definitions (i) and (ii), the number of predicted aftershocks becomes quite stable for $n_{real} \geq 10$ but the best $\chi(T)$ value is an increasing function of n_{real}. Conversely, by construction, the definition (iii) provides a $\chi(T)$ which converges, for $n_{real} \to \infty$, to the absolute minimum value of $\chi(T)$. In the revised version of the manuscript we adopt this procedure and explicitly show (new suppl. Fig.1) that the number of predicted aftershocks is quite stable for $n_{real} \geq 10$. In this way we motivate our choice $n_{real} = 10$. Nevertheless we wish to stress that we have verified that using the average or the median (definitions (i) and (ii)) for a fixed n_{real}, produces similar results with differences in the predicted aftershock number within the 20% of uncertainty.

Also this point is explicitly discussed in the Methods section of the revised version.

2a) The reviewer writes: "I was unable to reproduce a key figure. Using the parameter values in Table S2 I was unable to reproduce the predictions in Table S1 and figure 5. I came to my predicted numbers by integrating the Omori-Utsu law (Eq. 1) over the specified test interval/forecast window. I note that there are some entries in Table S1 that seem likely to be typos, and there is some confusion in the table caption."

We are very grateful to the reviewer for finding our mistake, now fixed in the revised version. The number of predicted aftershocks, in the interval $[t_{in}, t_f]$

Table 1: **Old table with the wrong parameters K and c.** For each main-shock we consider three different learning periods $T = 20min, 1h, 2h$.

**Code Name**	$K(T = 20min)$	$c(T = 20min)$	$K(T = 1h)$	$c(T = 1h)$	$K(T = 2h)$	$c(T = 2h)$
Crete	0.028	22582	0.016	22668	0.015	7937
Lixouri1	0.53	5755	0.77	23445	0.76	19971
Lixouri2	0.80	20386	0.55	10024	0.66	4938
North Aegean	0.16	3203	0.19	1280	0.17	773
Karpathos	0.38	34288	0.23	15084	0.218	12824
Lefkada	0.18	6765	0.22	16626	0.26	19084
Lesvos	0.56	71978	0.17	8000	0.18	4355
Kos	1.04	8567	1.00	3693	1.08	6342

is given by the integral of the Omori-Utsu law (Eq.1) which gives $n_{pred} = \frac{K10^{b\Delta m}}{p-1}\left((t_{in}+c)^{1-p} - (t_f+c)^{1-p}\right)$, with $\Delta m = 3$. The problem has been that, in a previous version of the manuscript, we used a 'normalized' Eq.(1)

$$\lambda(t) = \frac{(p-1)b\log(10)}{c^{1-p}}\frac{K_2 10^{b\Delta M}}{(t+c)^p}.$$

The result listed in the old Table S1 was for K_2 defined in the above equation and not for K of Eq.(1) in the manuscript. Moreover there is a further mistake in the definition of c which is expressed in numerical units instead of converting it in seconds. More precisely, in numerical simulations, we evaluate the synthetic envelope every 0.05 seconds. At the end the value of c listed in the old table S1 was in numerical units and to express it in seconds it is necessary to multiply those values by 0.05. In the revised version of the manuscript we have corrected both mistakes and in the new Table (now Table 3 in the main text) we insert the corrected values of both quantities K and c and we introduce the explicit formula for n_{pred}. We wish to emphasize that, since we have changed the inversion procedure (see answer 1c), these values are slightly different from the one of the previous version. For the sake of clarity below we report the old table S1 (with the wrong values) and the same table but with the corrected K and c values, obtained multiplying c by 0.05 and K by $\frac{(p-1)log(10)}{c^{1-p}}$. We also draw to the referee attention that t_f is not exactly equal to 3 days but to the final time of the recorded envelope.

We thank again the reviewer for pointing to our attention our mistake.

2b) The reviewer writes: " I'm quite confident that you mean a test period of [1h, 3days] and a learning period of [20min,1h,2h], but then your longest learning period overlaps with your test period. Was this intentional?"

Yes, this was intentional since gives us the opportunity to have a single value of n_{obs} independently of the learning period T. This allows us to use a single line to plot n_{obs} in Fig.5a that, from our point of view, makes the comparison between prediction and observation more simple to graphic. This has also the

Table 2: **Old table with the correct parameters K and c.** For each mainshock we consider three different learning periods $T = 20min, 1h, 2h$.

**Code Name**	$K(T = 20min)$	$c(T = 20min)$	$K(T = 1h)$	$c(T = 1h)$	$K(T = 2h)$	$c(T = 2h)$
Crete	0.013	1129	0.007	1133	0.006	396
Lixouri1	0.21	287	0.36	1172	0.35	998
Lixouri2	0.36	1019	0.23	501	0.26	247
NorthAegean	0.064	160	0.066	64	0.056	39
Karpathos	0.18	1714	0.10	754	0.096	641
Lefkada	0.07	338	0.099	831	0.11	954
Lesvos	0.29	3598	0.071	400	0.071	217
Kos	0.43	428	0.39	185	0.44	317

advantage of making clearer the fluctuations in the inverted parameters, as function of T. This choice is justified by the fact that we are mainly interested in the forecasting at short times $T \leq 1$h where the overlap, between learning and testing periods, is null. Furthermore we typically find that the agreement between n_{pred} and n_{obs} becomes worst for increasing $T > 2$h and therefore there is no practical advantage in the overlap between the two periods. However, if the reviewer judges our choice confusing we can modify it.

2c) The reviewer writes:"I get forecast numbers that are roughly twice those reported using the specified parameters."

This observation is a consequence of our mistake and is confirmed by the wrong values of K (reported in Table 1 of this answer) which are roughly twice the correct values of K (reported in Table 2).

2d) The reviewer writes: "Anwyay, the claim in the abstract of the forecast always being with 20% seems erroneous, given a quick glance at the numbers in Table S1. Is this value meant to apply only to a certain learning and test interval?".

The value of 20% was a rough estimate obtained using the optimal value in the interval $[0.5, 2]$h for each earthquake. In the abstract of the revised version we now give the maximum discrepancy estimated at $T = 1h$.

2e) The reviewer writes:"The authors should comfirm their calculations and check that all of the Omori parameters are correctly reported in Tables S1 and S2, as well as elsewhere in the manuscript (e.g. b=1, p=1.1)."

We have corrected the mistake and checked the new Omori parameters. We have also moved Supplementary tables in the main text and specified in the text and in the Table caption that we use the values $b = 1$ and $p = 1.1$.

3a) The reviewer writes: "It seems this paper could benefit from a more comprehensive analysis, using more data. Why only look at the closest station? The closest station is likely to be clipped, and additional stations would bring

in additional data, allowing you to assess the precision and robustness of your method on a distribution of station distances for a single event."

We thank the reviewer for this useful suggestion which we took carefully into account in the revised version of the manuscript where we also present, for the same earthquake, the envelope $\overline{\mu}(t)$ of the signal recorded at more than one station with different epicentral distance δr. In the manuscript we plot results for the recent 2018 Zakynthos earthquake and for the 2017 Kos and 2013 Crete earthquakes representing the two earthquakes with the largest and smallest number of aftershocks, respectively. This study (new Fig.4) clearly shows that, as expected, by increasing δr the perceived magnitude μ_M decreases whereas τ_M increases. However, after the vertical shift $\overline{\mu}(t) - \mu_M$ and the time rescaling $(t - t_0)/\tau_M$, data collapse is observed (new Fig.4) up to the time when the background contribution μ_B (Eq.4) becomes relevant and a flat behavior is observed. Obviously, the smaller the difference $\mu_M - \mu_B$ the smaller is the temporal regime where $\overline{\mu}(t)$ is dominated by the aftershock occurrence ($\overline{\mu}(t) \gg \mu_B$) and the less accurate is the estimate of the Omori parameters. The same behavior is found for all the earthquakes and motivates our choice to invert parameters using the signal from the closest station. We deeply discuss this point in the revised version.

3b) The reviewer writes:"This seems all the more important given that you are using only 8 area earthquakes. Why only look at 8 regional earthquakes in the first place? You are making the case that you have discovered somethIng fundamentally new and useful, but only demonstrating it with a bare handful of earthquakes."

We thank the reviewer for considering our findings "fundamentally new and useful". We have preferred to work with a limited number of earthquakes but selected according to a transparent criterion instead of considering a larger sample but with an AD HOC selection. Our criterion is to consider a geographic region where we can have easily access to all recorded waveforms and to consider all the earthquakes, above a given threshold, occurred after a given time. In this way we obtained a small but significant statistical sample which includes 8 earthquakes occurred in different tectonic environments (see Fig.1). We have verified that our method properly works for ALL the 8 earthquakes.

Nevertheless, stimulated by the reviewer's observation and in order to be coherent with our adopted criterion, in the revised version, we test our method also for the M=6.8 Zakynthos earthquake occurred on 25 October 2018, after our first submission. Our method provides very satisfactory result also for the new earthquake. This, from our point of view, strongly supports our procedure, not so much for the increased testing sample (from 8 to 9) but because the novel analysis can be considered as a prospective test of our method. In the revised version of the manuscript we present the results applied to the recent Zakynthos earthquake.

4a) The reviewer writes: "You observe that the best agreement is obtained for a learning period less than 2h. You attribute this to secondary triggering.

I find this very confusing. Your forecast should get worse with time away from the learning period due to secondary triggering, but it makes no sense that extending the learning period should degrade the forecast, precisely because the longer learning period captures more of the secondary triggering."

As shown in Helmstetter and Sornette (2002) secondary triggering significantly affects statistical features of aftershocks for times larger than a characteristic time t^*. For instance, the p-value of the Omori law changes from $p = 1 + \theta$ with $\theta > 0$ when $t < t^*$ to $p = 1 - \theta$ when $t > t^*$. As a consequence, since we consider a single Omori law, we expect to obtain the correct Omori parameters K and c only for learning periods $T < t^*$. Conversely, when $T > t^*$ the Omori parameters must be evidently wrong. Since it is reasonable to expect that the majority of our target aftershocks ($\mu_i > \mu_M - 3$) has been directly triggered by the mainshock, it is also reasonable that the fit with a single Omori law, with the correct parameters, gives satisfactory results. Conversely, the behavior at large T suggests that our inversion procedure does not adapt to properly capture secondary triggering leading to a less accurate aftershock forecasting. However this is only a possible interpretation. We try to better explain this interpretation in the revised manuscript.

4b) The reviewer writes: "I also do not buy the explanation that the large misfit for the N. Aegean sequence can be explained by the change in slope. The slope change is *very* subtle, and the same change in slope is present in the Kos data."

We have checked this point by considering $\overline{\mu}(t)$, for both the Kos and N. Aegean earthquakes, over a longer temporal interval (5 days). We are compelled to agree with the reviewer that the change of slope is similar in both earthquakes and therefore it is difficult to attribute the very fast decrease of the number of aftershocks after the N. Aegean earthquake to the observed change of slope in $\overline{\mu}(t)$. We have removed this comment in the revised version.

5a) The reviewer writes: "Presentation: There are a number of typos in this mansucript, including in the figure legends."

We have double–checked through the entire manuscript and corrected typos.

5b) The reviewer writes: "Many of the figures are hard to read. For example, it is very difficult to tell open and filled symbols (data and model) apart in figure 2."

Fig.3 contains only instrumental data plotted as filled symbols. In the revised version of the manuscript we have clarified this point and improved the data style.

5c) The reviewer writes: "I also find it very difficult to see all of the symbols in Figure 5."

We have split the two panels of the old Figure 5 into two separate figures. In the Supplementary materials we also present the zoom of different regions of the old Figure 5.

Reviewers' comments:

Reviewer #2 (Remarks to the Author):

I still think this study is a great idea, but I am surprised to find that I still cannot reproduce the numbers in Table 2. (I stopped after checking only one earthquake, though: the short time forecast for the North Aegean.) The issues mentioned below may be contributing factors. Right now I can't say whether the results are meaningful or not, because the testing window is not clearly defined, and I can't reproduce the numbers. These issues could be minor, or they could represent something more fundamental.

1) Table 2 still erroneously identifies the learning and testing periods in the caption.

2) The paper nowhere discusses the overlap between the learning and testing windows. Since aftershock activity is highest in the earliest part of the testing window, it is impossible to judge how much of the prediction success is gotten "for free." Please start the testing period at 2hrs for all learning intervals, if you want to use a single consistent testing period for all learning periods.

Also not discussed in the paper is the fact mentioned in the rebuttal, that the testing window is not necessarily as claimed in the paper, but rather extends only to the "final time of the recorded envelope." What is this time? For which earthquakes do you lack instrumental data? If the testing interval is not actually 3 days, then the results are not as compelling as claimed, but it would explain why it is impossible to check the numbers.

3) Please list the units of c in Table 3 for reproducibility.

"Answer to the 2nd report of Reviewer #2"

We thank the reviewer for the further reading of the manuscript and for considering our study a "great idea". We regret that s/he "still cannot reproduce the numbers in Table 2. (I stopped after checking only one earthquake, though: the short time forecast for the North Aegean.)". In the first version of the manuscript the problem was caused by our erroneous definition of the parameters K and c and we are still very grateful to the reviewer for drawing our attention to it. In the second version, conversely, we have chosen to present the values of K with only three digits and this causes differences with the values of $n_{exp}(T)$ reported in Table 2. These differences, however, were always smaller than the 10% and therefore well within the error bars. Nevertheless, following the reviewer's remark, we have included more digits in the revised version in order to allow the reader to recover the exact values listed in the table. As an example, let us consider the North Aegean earthquake for $T = 20$ min, in the old Table 3 we had $K = 0.07$ and $c = 281$ sec and the time window was $[t_{in} = 1h, t_f = 3days]$. A key point is to express time (t_{in}, t_f, c) in seconds (see point 3 in this answer). The quantity

$$n_{pred}(T) = \frac{K10^{b\Delta M}}{p-1}\left((t_{in}+c)^{1-p} - (t_f+c)^{1-p}\right),$$

with $\Delta M = 3$, $b = 1$ and $p = 1.1$ reads $n_{pred}(T) = 105$ different from $n_{pred}(T) = 114$ reported in the Old Table 2, now Supplementary Table 1. Conversely the value $K = 0.0757$, now listed in the new Table 3, correctly leads to $n_{pred}(T) = 114$. We have double-checked that all values of $n_{pred}(T)$ listed in the new Table 2 are exactly obtained from the corresponding values of K and c listed in Table 3.

In the following we answer to the specific remarks:

1) The reviewer writes: "Table 2 still erroneously identifies the learning and testing periods in the caption."

The reviewer is right. We have corrected it in the revised version of the manuscript.

2a) The reviewer writes: "The paper nowhere discusses the overlap between the learning and testing windows."

We discuss the presence of the overlap in the revised version.

2b) The reviewer writes:"Since aftershock activity is highest in the earliest part of the testing window, it is impossible to judge how much of the prediction success is gotten "for free." Please start the testing period at 2hrs for all learning intervals, if you want to use a single consistent testing period for all learning periods."

We thank the reviewer for this suggestion which we have followed in the revised version of the manuscript. More precisely, the new Fig.6, the new Table 2 and the new Suppl. Fig.2 present results for the testing period $[t_{in} = 2h, t_f = 3days]$. Previous conclusions, obtained for the case with $t_{in} = 1h$, are still valid. For the sake of completeness, the old results for $t_{in} = 1h$ have been moved to the novel Supplementary Materials.

2c) The reviewer writes: "Also not discussed in the paper is the fact mentioned in the rebuttal, that the testing window is not necessarily as claimed in the paper, but rather extends only to the "final time of the recorded envelope." What is this time? For which earthquakes do you lack instrumental data? If the testing interval is not actually 3 days, then the results are not as compelling as claimed, but it would explain why it is impossible to check the numbers."

The final time t_f of the testing period is always 3 days except for the Crete earthquake ($48h$) and the Karpathos earthquake ($56h$). This difference in the t_f values produces small differences in the value of $n_{pred}(T)$ and we did not mention it in the previous version. However, in the resubmitted version of the manuscript we explicitly gives the values of t_f for the Crete and Karpathos earthquake in the captions of Fig.6 and Table 2.

3) The reviewer writes: "Please list the units of c in Table 3 for reproducibility."

We thank the reviewer for highlighting this missing information which is now present in the caption of Table 3.

Reviewers' comments:

Reviewer #2 (Remarks to the Author):

I appreciate seeing the second revision of this manuscript. The authors have added the requested information, and I can reproduce the numbers in table 2, now.

Digging a little deeper, though, however, I find another alarming feature: the model fails badly to predict the number of earthquakes between 1 and 3 months given in table 1. Only about 40% of the earthquakes in the interval between 7 days and 3 months should occur after the first month, so I am surprised that the performance should degrade so dramatically. The error is 2-3 orders of magnitude in some cases, well outside the "typical" 10% accuracy reported. I have not gone so far as to download the Greek earthquake catalog and confirm the observed numbers from 7 days to 3 months shown in figure 7. The authors should address the breakdown of the forecast between 1 and 3 months, or explain why the numbers in the Table 1 are irrelevant.

Finally (and I commented on this in the first round) it strikes me that the statement of the forecast having a "typical" accuracy of 10% is only about 30% accurate, in that only 1/3rd of the 1hr - 3day forecasts meet this level. Likewise, the maximum misfit is closer to 40% than 30%. These are fine numbers in aftershock forecasting. Why exaggerate?

"Answer to the 3rd report of Reviewer #2"

We thank the reviewer for further reviewing our manuscript and for pointing to our attention the problem with the values of N_1 and N_3 listed in Table 1. In the following we answer to each specific observation.

1) The reviewer writes:

"Digging a little deeper, though, however, I find another alarming feature: the model fails badly to predict the number of earthquakes between 1 and 3 months given in table 1. Only about 40% of the earthquakes in the interval between 7 days and 3 months should occur after the first month, so I am surprised that the performance should degrade so dramatically. The error is 2-3 orders of magnitude in some cases, well outside the "typical" 10% accuracy reported. I have not gone so far as to download the Greek earthquake catalog and confirm the observed numbers from 7 days to 3 months shown in figure 7. The authors should address the breakdown of the forecast between 1 and 3 months, or explain why the numbers in the Table 1 are irrelevant."

The old Table 1 was compiled implementing the information extracted from previous studies and, in particular, the definition of aftershocks does not exactly correspond to the one adopted in the present study. More precisely, here we define aftershocks as events occurring within a radius $L_M = 0.02 \times 10^{0.5 m_M}$ from the mainshock epicenter whereas, in the old Table 1, aftershocks are events occurring within a box of 25 km centered in the mainshock epicenter. The two definitions produce small differences of the order of 10%. The real problem was with the Lefkada, Lesvos and Kos mainshocks, since the values of N_1 and N_3 were extracted from the less accurate USGS catalog and this gives values significantly smaller than those found in the official Greek catalog.

In the Table 1 of the revised version we use a definition of aftershock number coherent with the rest of the paper and we always use the information from the Greek catalog. We also add the novel suppl.Fig.5 where we explicitly compare the number of predicted aftershocks in the temporal window $[1, 3]$ months with the number recorded in the Greek catalog. This figure shows a reasonable agreement (up to 20%) for the majority of mainshocks with much larger discrepancy (up to 60%) for the Kos mainshock and even worst for the North Aegean earthquake, when no aftershock has been recorded.

In the revised version we explicitly add the link to the Greek catalog, so that readers can directly verify the reported numbers.

2) The reviewer writes: "Finally (and I commented on this in the first round) it strikes me that the statement of the forecast having a "typical" accuracy of

10% is only about 30% accurate, in that only 1/3rd of the 1hr - 3day forecasts meet this level. Likewise, the maximum misfit is closer to 40% than 30%. These are fine numbers in aftershock forecasting. Why exaggerate?”

In the revised version we have changed the last sentence of the abstract to specify that we obtain an accuracy smaller than 18% for 8 earthquakes and the worst misfit is 36% for the Lefkada earthquake.

Reviewers' comments:

Reviewer #2 (Remarks to the Author):

To reiterate: the main issue I now have is that the model does not forecast the number of earthquakes between 1 and 3 months, despite claims in the paper that the numbers between 7 days and 3 months are forecast to 30% within a few minutes of the mainshock.

The observed numbers between 1 and 3 months from Table 1 are:
[2, 62, 65, 0 , 35, 3, 2, 5, 0]
The predicted numbers for (20 minutes, 1hr, and 2hr training windows are:
[2, 30, 33, 18, 18, 13, 11, 58, 56]
[2, 75, 49, 20, 23, 20, 20, 119, 91]
[2, 77, 61, 16, 23, 23, 22, 146, 99]

In absolute percent error, this is:
[19, 51, 59, INF, 50, 321, 445, 11, INF]%
[0.2, 22, 25, INF, 35, 557, 855, 2285, INF]%
[9, 24, 6, INF, 35, 673, 951, 281]%

These numbers greatly exceed the stated 10% typical error. Can the authors justify this discrepancy? At this point, I must admit that I am unlikely to be convinced by the manuscript, in that the model is now in the unfortunate position of being tuned specifically to fit the comments of this reviewer. Unless the authors can point out the error in my analysis, I would not recommend publishing this paper.
