# Supplementary material for: Forecasting of the first hour aftershocks by means of the perceived magnitude
Source: Nat Commun. 2019 Jul 4;10:2953. doi: 10.1038/s41467-019-10763-3 (PMC6609666; doi:10.1038/s41467-019-10763-3)
Supplement: Supplementary file 1 — Peer Review File [file 41467_2019_10763_MOESM1_ESM.pdf]

Supporting Information for
"Forecasting of the first hour aftershocks by means of the perceived magnitude"

Lippiello E.[1], Petrillo G.[1], Godano G. [1], Tramelli A. [2], Papadimitriou E. [3], Karakostas V. [4]

[1]Department of Mathematics and Physics and CNISM, Second University of Naples, 81100 Caserta, Italy.

[2]INGV, Napoli, Italy

[3]Geophysics Department, Aristotle University of Thessaloniki GR 541 24 Thessaloniki Greece

Supplementary Table 1. The comparison between the observed n_{obs} and predicted $n_{pred}(T)$ aftershocks for $t_{in} = 1$h. The same of Table 2 of the manuscript with a testing period with $t_{in} = 1$h. The quantity $n_{pred}(T)$ is obtained with the values of K and c listed in Table 3 of the manuscript and using $b = 1$ and $p = 1.1$.

**Code Name**	n_{obs}	$n_{pred}(20min)$	$n_{pred}(1h)$	$n_{pred}(2h)$
Crete	13	15	12	13
Lixouri1	490	193	481	487
Lixouri2	298	213	310	391
North Aegean	117	114	126	100
Karpathos	133	106	135	136
Lefkada	188	80	125	148
Lesvos	138	71	123	136
Kos	663	372	768	937
Zakynthos	512	357	580	634

Corresponding author: E. Lippiello, `eugenio.lippiello@unicampania.it`

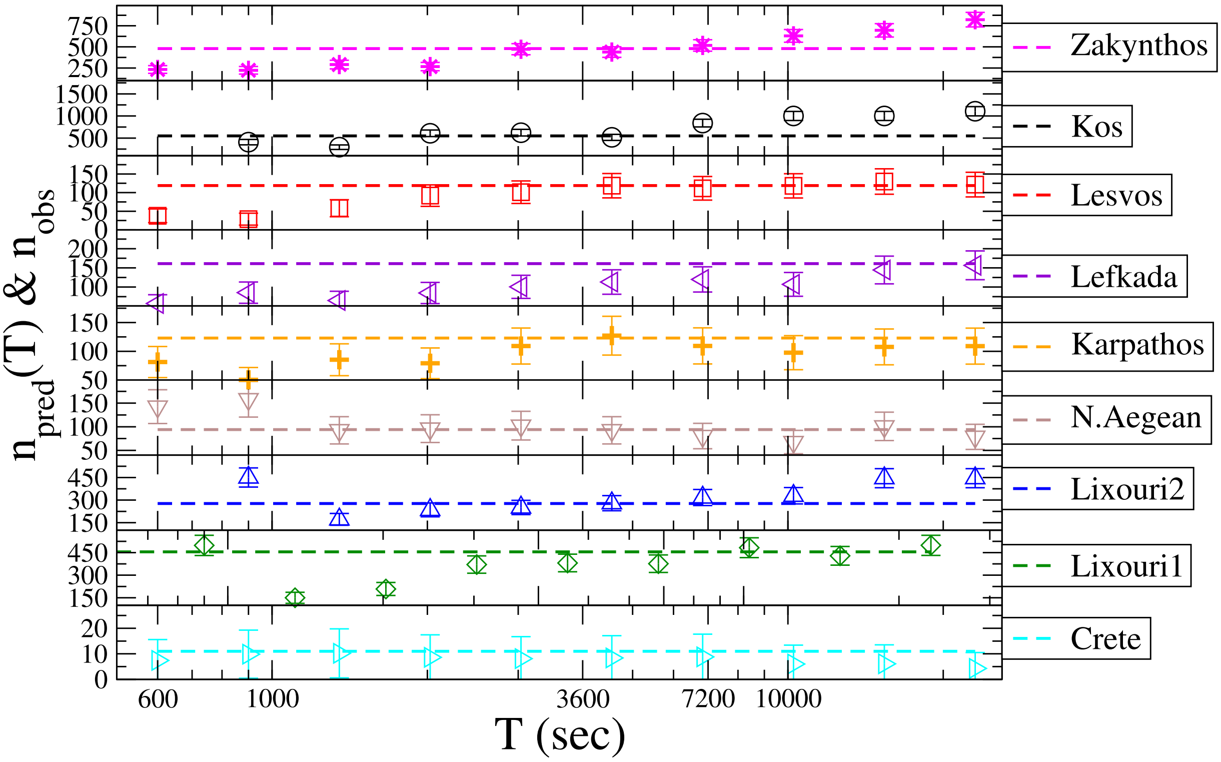

Supplementary Figure 1. Short-term test of the method for the recent Aegean earthquakes. The same of Fig.6 of the manuscript with linear scale on the vertical axis and a zoom on each main-aftershock sequence.

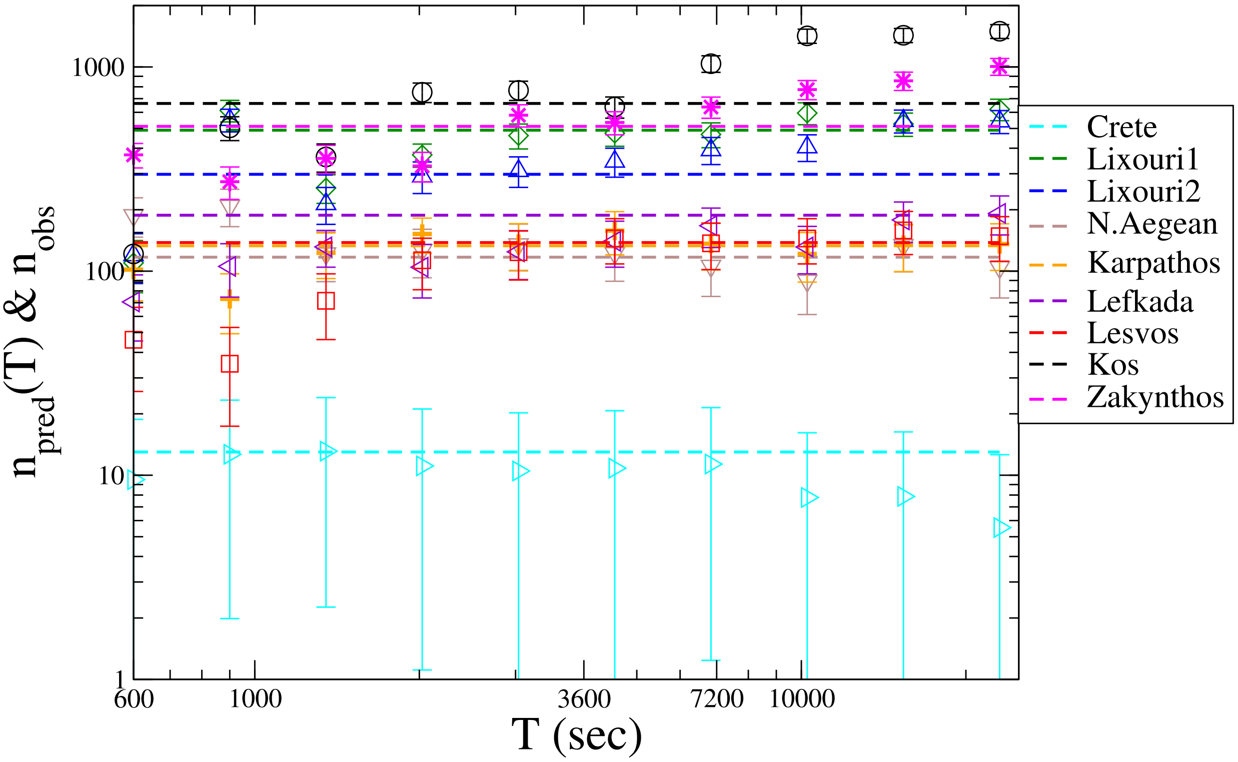

Supplementary Figure 2. Short-term test of the method for the recent Aegean earthquakes for t_{in} = 1 h. The same of Fig.6 of the manuscript for a testing period with $t_{in} = 1h$.

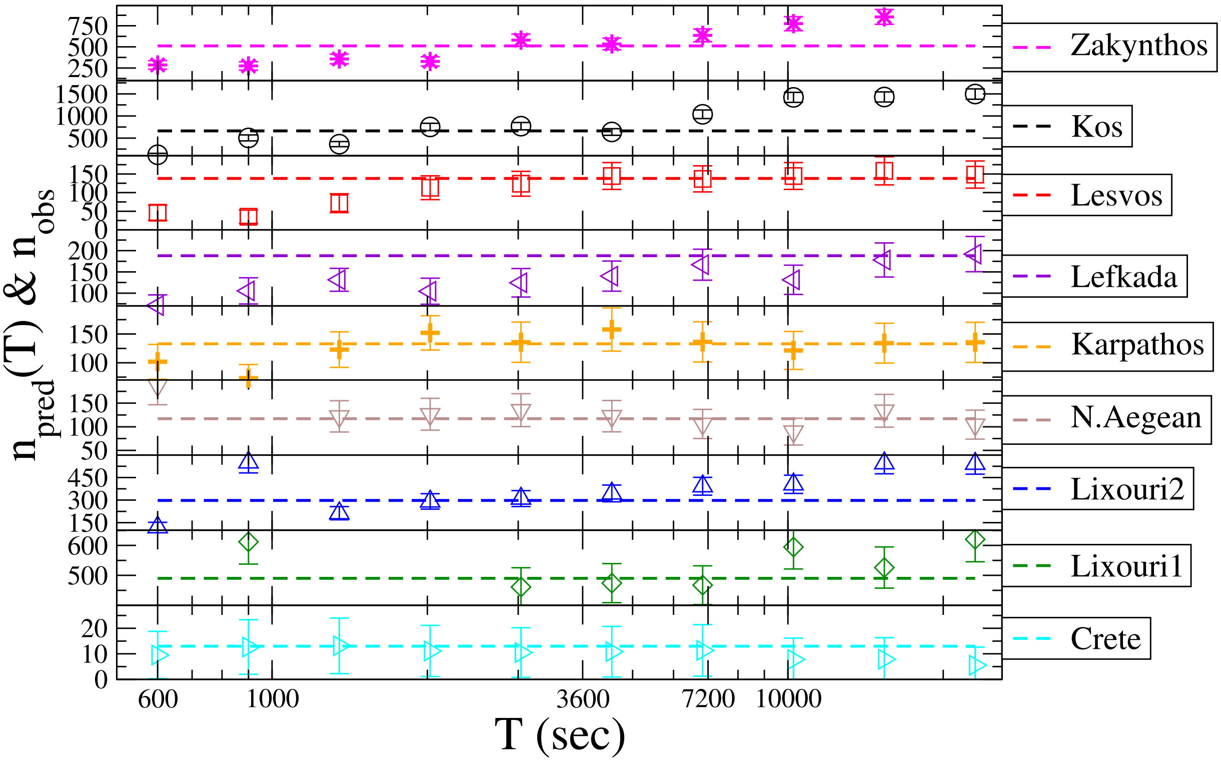

Supplementary Figure 3. Short-term test of the method for the recent Aegean earthquakes for $t_{in} = 1h$. The same of Supplementary Figure 1 with a testing period with $t_{in} = 1h$.

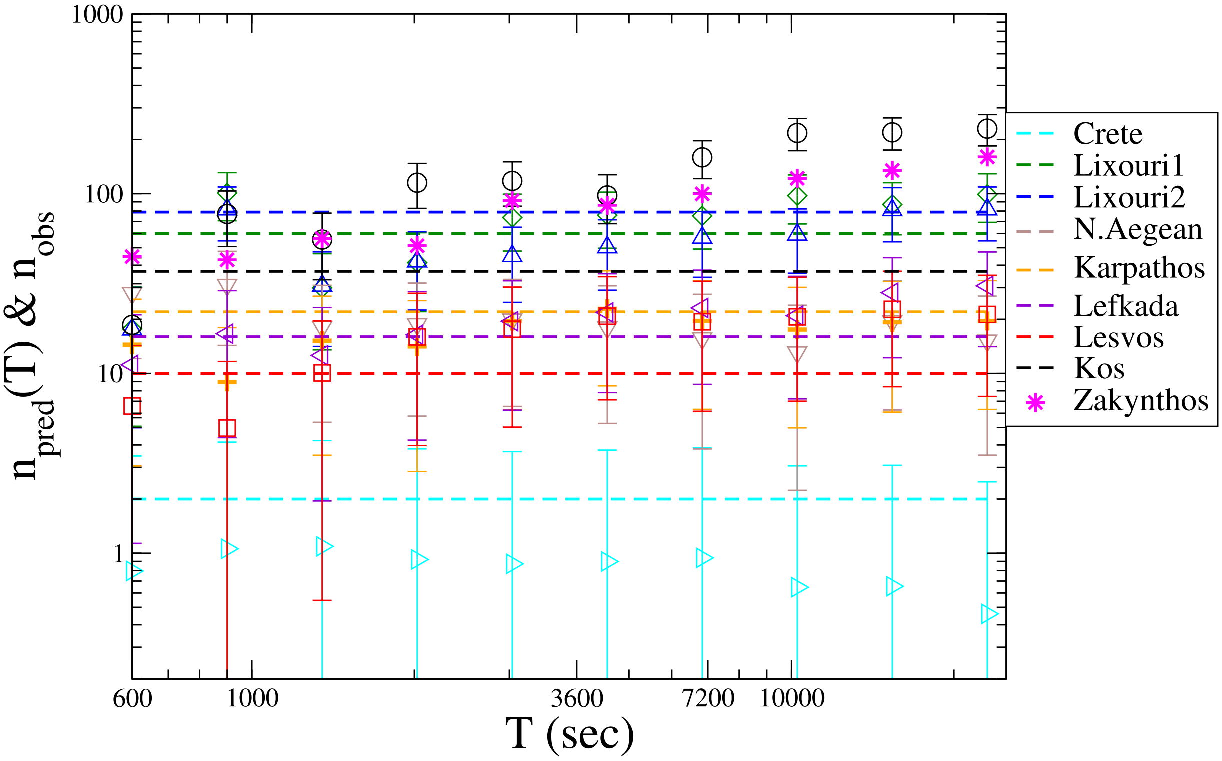

Supplementary Figure 4. Long-term test of the method for the recent Aegean earthquakes. The same of Fig.7 of the manuscript, for the number of predicted aftershocks $n_{pred}(T)$ (open symbols) with $\mu_i > \mu_M - 3$ in the testing period $[30, 90] days$. Dashed horizontal lines is the number of aftershocks with $m_i > M_m - 3$ recorded in the Greek catalog in the interval $[30, 90]$ days. Aftershocks are defined as events occurring within a radius of size $L_M = 0.02 \times 10^{0.5 m_M}$ from each mainshock epicenter.

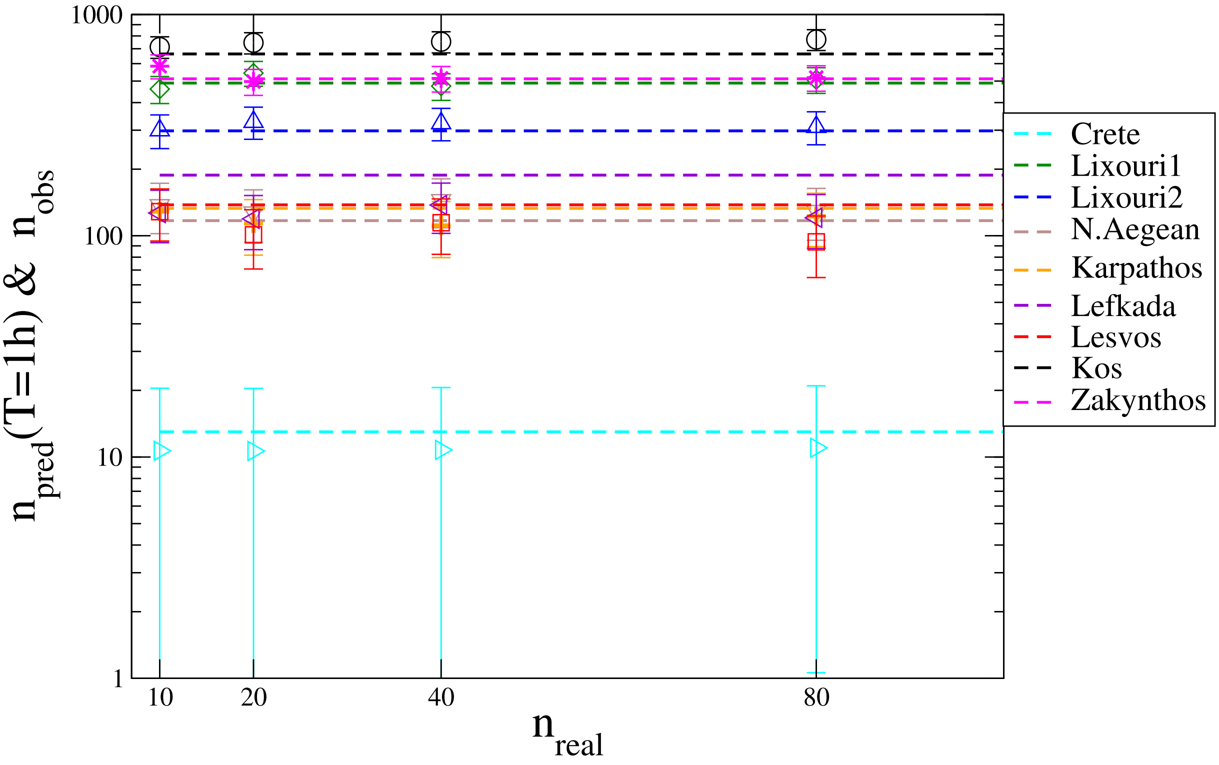

Supplementary Figure 5. Test of the method for the recent Aegean earthquakes for different values of n_{real}**.** The number of observed aftershocks with $\mu_i > \mu_M - 3$ in the testing period $[t_{in} = 1h, t_f = 3days]$ is plotted with dashed horizontal lines with different colors for different mainshocks (see legend). Open symbols represent the value of $n_{pred}(T)$, obtained with our method, for different values of the number of independent realizations n_{real} and for a testing period $T = 1h$. The error bars correspond to statistical fluctuations in the number of expected aftershocks for fixed K and c values.
